# *Minimal inhibitory concentration* of microorganisms causing surgical site infection in referral hospitals in North of Iran, 2011-2012

**Published:** 2015

**Authors:** Ahmad Alikhani, Farhang Babamahmoodi, Laleh Foroutan Alizadegan, Arman Shojaeefar, Abdolreza Babamahmoodi

**Affiliations:** 1 Department of Antimicrobial Resistance Research Center, Faculty of Medicine, Mazandaran University of Medical Sciences, Sari, Iran.; 2 Mazandaran University of Medical Sciences, Sari, Iran.; 3 Ghaemshahr Razi Hospital, Ghaemshahr, Iran.; 4 Health Management Research Center, Baqiyatallah University of Medical Sciences, Tehran, Iran.

**Keywords:** Minimal Inhibitory Concentration, Surgical site infection, Resistance, Antibiotics

## Abstract

**Background:**

A surgical site infection (SSI) is the most common nosocomial infection after surgery and is the third most common infection in hospitalized patients. The aim of this study was to asses minimum inhibitory concentration (MIC) of the causing agents of SSI and antimicrobial susceptibility patterns.

**Methods:**

This cross-sectional study was done in three referral hospitals in North of Iran during 2011-2012. The samples were taken one month after orthopedic, abdominal, cesarean section surgery and *coronary artery bypass graft* (CABG) in patients with scores compatible to SSIs criteria. The sample was sent for bacteriologic culture and MIC determination for positive cases by broth microdilution method. The data were collected and analyzed.

**Results:**

From 103 positive cases S. aureus, E.coli and coagulase negative staphylococci were the most common isolated agents as 29.12%, 23.3% and 21.3%, respectively. S. aureus was sensitive to vancomycin (70%), amikacin (70%) and teicoplanin (76.6%) and cogulase negative staphylococci was sensitive to vancomycin (68.1%) and teicoplanin (72.6%) and E.coli to amikacin (95.83%) and imipenem and meropenem (66.66%). P.aeroginosa showed no sensitivity to cefepime and was sensitive to imipenem (93.75%) and meropenem (81.25%).

**Conclusion:**

The most important point is worrisome problem of the increased MIC of S. aureus to vancomycin that causes difficult use in the treatment of staphylococcal SSIs. In spite of resistance of micro-organisms to cephalosporins, gram negative organisms had low MIC to carbapenemes especially P.aeroginosa although the rate of its MIC is increasing.

Ahospital-acquired infection (HAI) is an infection whose development is favored by a hospital environment induced by pathogenic reactions related to infectious agent itself or its toxins, provided that it is induced 48 to 72 hours after admission and that the patient has no signs of the infection at the time of admission, or the disease is in incubation period (1). According to the studies conducted by the World Health Organization (WHO) in 2005, more than 1.4 million of people in the world suffered from hospital-associated infections (2).

Surgical site infection (SSI) is the most common nosocomial infection developed by those patients who underwent surgery, and constitutes the third common infection acquired in hospital (3). SSI increases hospitalization period from 7.4 days to 14.3 days in the hospital (4). In spite of all attempts made to prevent SSI, this problem continues to have high prevalence. According to the information obtained from an analysis conducted by National Health Statistics Center and National Healthcare Safety Network (NHSN), almost among 26.6 millions of hospitalized patients who undergo surgery annually, between 250,000 to 1,000,000 of SSI occur (2). A more current concern is the increase in bacteria that show resistance to important antibiotics. 

There is great concern about the emergence of S. aureus with reduced susceptibility to vancomycin due to the high incidence of the organism in causing both health care- and community-associated infections and its well-known virulence and resistance to many other antimicrobial agents (5, 6). Accordingly, this study was designed and conducted to identify SSI inducing microorganisms and their antimicrobial susceptibility pattern using MIC determination.

## Methods

In this cross-sectional study, the patients who were admitted to Razi Hospital in Ghaemshahr City, Imam Khomeini Hospital and Fatemeh Zahra Hospital in Sari city in 2011 and in the first 6 months of 2012 who underwent abdominal surgery, orthopedic, open heart surgery, and cesarean section (CS) with SSI criteria, were studied. Accordingly, the inclusion criteria were discharge of pus from the incision site, infectious wounds which were reopened according to the surgeon’s diagnosis, due to discharge (serous or purulent discharge), existence of serous or non-purulent discharges along with signs of inflammation such as edema, redness, warmth, stiffness, and tenderness and infection of the wounds, induced 30 days after surgery and, in case of implant, through a year after (4). The exclusion criterion include patients with infectious wounds not induced by surgery.

In the present study, surface infections was defined as purulent or non-purulent discharges from the incision site along with signs of inflammation, edema, redness, warmth, stiffness and tenderness. In these cases, for the purpose of preparing sample from exudates, without washing, the sampling was made using two sterile swabs, one was sent to the laboratory for preparing smear and staining and the other one for culture in terms of Gram-positive cocci, Gram-negative bacilli, and pseudomonas, without the need for transport environment. If infections occurred in deeper tissues, and some evidence in favor of pleural effusion was confirmed by sonography, first the related site was sterilized by alcohol and then, the sterile needle was inserted to the site, the discharge was aspirated into the sterile syringe and sent to the laboratory. In the laboratory, the first swab was cultured in the medium of Nutrient Broth and the second swab in the media of Eosin Methylene Blue (EMB), blood agar, and chocolate agar.

Then, the media were put in the incubator (37ºC) for 24 hours. After they are exited from the incubator, if the content of the Nutrient Broth became dark, this indicated growth of bacteria, and then it was passed to the media of EMB, blood agar, and chocolate agar for the purpose of preparing isolated colony, and placed in the incubator for 24 hours again. After that, the bacterial colony grew, a smear was prepared from it, stained and was examined by microscope in terms of the shape of microbes (bacilli or cocci) and Gram staining. Catalase test, coagulase test, oxidase test, lactose fermentation test, indole test, urease test, VP MR and citrate tests were used to identify bacterial species. The bacterial suspension equivalent to 0.5 McFarland standard was prepared, the number of bacteria was 10(8)×1.5 CFU / ml. then, the final standard concentration of the bacteria was increased to 5×10^5^ CFU / ml. This standard suspension was used to determine MIC compared to the antibiotics of ceftriaxone, amikacin, oxacillin, vancomycin, meropenem, imipenem, cefepime, teicoplanin, ciprofloxacin (7). The collected information was encoded and the obtained data were statistically analyzed by the statistical software of SPSS 19. The findings obtained from this study were interpreted in accordance with the standard table of (M100-A21) 2011 CLSI (8). P<0.05 was considered significant statistically.

## Results

The number of participants in this study was 103 SSI cases of whom 60% (62 cases) were men and 40% (41 cases) were women and the age range was 34-84 years with a mean age of 34+50 years. A review of these patients showed that 41.7% of them (43 cases) had a history of systemic disease that included high blood pressure in 10 cases (9.7%), diabetes in 9 cases (8.7%), malignancy in 7 cases (9.7%), diabetes and hypertension in 10 cases (9.7%), renal failure and diabetes in 9 cases (1.9%) anemia in 5 (4.8%) and among these cases, 25 had emergent and 78 elective surgery. In our cases, 27 (26.2%) patients had orthopedic surgery, 12 (11.6%) gynecological, 10 (9.7%) head and neck, 6 (5.8%) vascular, 6 (5.8%) open heart, 4 (3.8%) thoracic, and 38 (36.8%) with various general surgeries of whom out of these, 13 (34.2%) had appendix surgery, 8 (21%) intestinal, 6 (15.7%) gallbladder, 6 (15.7%) herniorrhaphy, and 5 (13.1%) gastric surgery.

**Type of microorganisms isolated from SSI:** A total of 103 positive media were surveyed,s.aureus with 30 (29.12%) cases, E.coli 24 (23.3%), and coagulase negative staphylococci 22 (21.3%) cases. They were among the agents isolated from the infection of the incision site in these patients.

**Determination of Sensitivity of Microorganisms (included intermediate level)**: The analysis of anti-microbial susceptibility of the isolated bacteria was made by determining MIC. The most common isolated agents were S. aureus which showed the highest sensitivity to teicoplanin (76.6%), amikacin (70%), and vancomycin (70%). This bacterium showed 100% resistance to oxacillin and ceftriaxone. The second common agent in this study was E.coli which showed the highest sensitivity to amikacin (95.86%), imipenem (66.66%) and meropenem (66.66%), but was resistant to cefepime (100%) and ceftriaxone (91.6%). The third common micro-organism was coagulase negative staphylococci with the highest sensitivity to teicoplanin (72.6%) and vancomycin (68.1%). The information for the sensitivity and MIC of all bacteria is shown in tables 1 to 4.

**Table 1:** Antibiotic susceptibility pattern of Staphylococcus aureus in surgical site infections

	**Breakpoint**		**S (%)**	**I (%)**	**R (%)**	**MIC50**	**MIC90**	**MIC range**
	**S**	**R**						
Cloxacilline	**<**2	**>**4	_	_	100	_	1024	128-1024
Amikacin	**<**16	**>**64	60	10	30	16	128	4-256
Cefepime	**<**8	**>**32	_	10	90	512	1024	4-1024
Vancomycin	**<**2	**>**16	50	20	30	2	32	0.5-128
Teicoplanin	**<**8	**>**32	60	16.6	23.4	4	64	0.5-128

**Table 2:** Antibiotic susceptibility pattern of coagulase negative staphylococcus in surgical site infections

	**S (%)**	**I (%)**	**R (%)**	**MIC 50**	**MIC90**	**MIC range**	**Breakpoint**	
							**S**	**R**
Vancomycin	54.50	13.60	31.90	4	256	0.5-512	<4	>32
Teicoplanin	59	13.60	27.20	8	1024	0.5-1024	<8	>32
Cefepime	9.1	0	90.9	-	1024	4-1024	<8	>32
Cloxacilline	13.60	0	86.30	-	1024	0.25-1024	<0.25	>0.50
Amikacin	4.50	9	86.3	-	256	16-1024	<16	>64
Ceftriaxone	0	0	100	-	1024	64-1024	<8	>64

**Table 3:** Antibiotic susceptibility pattern of Escherichia coli  in surgical site infections

	**S(%)**	**I(%)**	**R(%)**	**MIC50**	**MIC90**	**MIC range**	**Breakpoint**	
							**S**	**R**
Cefepime	0	0	100	-	256	64-512	<8	>32
Gentamicin	0	16.66	83.33	32	256	8-256	<4	>16
Meropenem	54.16	12.50	33.34	4	32	1-128	<4	>16
Imipenem	58.33	8.33	33.34	4	32	1-256	<4	>16
Amikacin	58.33	37.50	4.16	16	64	4-64	<16	>64
Ceftriaxone	8.33	0	91.67	512	1024	2-1024	<8	>64

**Table 4:** Antibiotic susceptibility pattern of pseudomonas aeroginosa in surgical site infections

	**S (%)**	**I (%)**	**R (%)**	**MIC50**	**MIC90**	**MIC range**	**Breakpoint**	
							**S**	**R**
Cefepime	0	0	100	-	1024	65-1024	<8	>32
Meropenem	75	6.25	18.7	4	32	1-128	<4	>16
Imipenem	87.5	6.25	6.25	4	8	0.5-64	<4	>16
Amikacin	43.75	0	56.25	8	128	2-128	<16	>64
Ceftriaxone	6.25	12.5	81.25	-	256	8-256	<8	>64

## Discussion

In the present study, of 103 positive culture surveyed, S. aureus with 30 (29.12%) cases, E. coli with 24 cases (23.3%), and coagulase negative staphylococci with 22 cases (21.3%) were among the isolated agents from the infection of the incision site in these patients. Similar to our study, in the studies conducted by Misteli in Switzerland, Rolston in the United States, and Afifi in Egypt, the most frequent microorganisms creating SSI was S. aureus. However, in the study conducted by Afifi, after S. aureus, pseudomonas and E. coli were the most frequent and coagulase negative staphylococcus ranked the third. While in Misteli’s study, Similar to our study, after S. aureus, E. coli with the frequency of 20.9% and coagulase negative staphylococci with the frequency of 12.4% were among the most common organisms isolated from the incision site (9-11). As shown by these studies, one of the most common organisms causing SSI was S. aureus for which first-generation cephalosporin or oxacillin and in cases of high resistance in hospitals, vancomycin is usually used. However, the results of our study showed that the above-mentioned antibiotics have no longer considerable effect on these microorganisms particularly vancomycin. As we have shown, about 30% of species of S. aureus were fully resistant to this antibiotic and 20% of the species showed intermediate resistance. After conducting a five-year study on resistance pattern of the species of S. aureus to vancomycin, Wang showed the decreased sensitivity of S. aureus to vancomycin faded over time on the effective role of this antibiotic as an appropriate coverage against Gram-positive cocci, because MRSA strains continuously were exposed to vancomycin (12).

In the study of Saderi in 2005 in Tehran, which was reported according to CLSI before 2007, 3.6% of cases were resistant to vancomycin (13). In the study conducted by Hadadi in Tehran (2009), four years after the study conducted by Saderi, no S. aureus was reportedly resistant to vancomycin (14). In the study conducted by Ali Gholi in Tehran (2008), two of 356 isolated staphylococcus aureus were resistant to vancomycin (15) but in Armin’s study in Tehran (2011), 30% of isolated S.aureus were resistant to vancomycin (16).

During 2005-2006, Khorvash showed that S. aureus and coagulase negative staphylococci were the most common agents of SSI and were sensitive to vancomycin completely (3). But this finding differs from the findings of our study. However, this study was conducted 7 years before our study began. During this period, a considerable change was made in CLSI criteria. According to UCLA manual and antimicrobial sensitivity tests in 2011, S. aureus has 99% sensitivity to vancomycin (17). So it seems that resistance to vancomycin occurs due to excessive administration of antibiotics. On the other hand, by the acquisition of resistance gene to vancomycin, these microorganisms become resistant to other antibiotics. This can be alarming, given the high frequency of S. aureus in creating hospital-acquired infections including SSI.As mentioned earlier, there are different reports from different studies conducted in several areas, but the important point is the increasing resistance of S. aureus to vancomycin.

To treat coagulase negative staphylococci, drugs such as vancomycin, teicoplanin, amikacin, and meropenem are suitable. In this study, 54.5% of them had full sensitivity to vancomycin, and 59% to teicoplanin. In the study conducted by Alikhani in 2008, 57.5% of them had full sensitivity to vancomycin. Given the similar findings, we found that the efficacy of vancomycin in covering all microorganisms is decreasing (18). Regarding E. coli, cephalosporins are not suitable drugs. In the study conducted by Khorvash, they showed 100% resistance to ceftriaxone and 76.5% to cefepime. This finding agrees with our findings (3). On the other hand, sensitivity of these agents in our study to imipenem and meropenem was 58.3 and 54.16%, respectively. It seems that amikacin at a sensitivitly of 66.6% can be considered a suitable alternative drug for treating SSI caused by E.coli, as a part of combination therapy.The mentioned findings indicate that although carbapenems are still suitable drugs for E.coli, but because of its drug resistance, still it covers likely. 

On the other hand, the carbapenems are also suitable drugs for covering pseudomonas, because in our analyses, 87.5% of pseudomonas's strain had full sensitivity to imipenem and 75% to meropenem. It should be noted that all cases of pseudomonas were resistant to cefepime. Given the similar findings in Rahimi's study in 2011 in Arak City on its 30% imipenem resistant (MIC ≥ 16), increasing resistance to imipenem among the strains of pseudomonas aeruginosa should be pointed. This will be an alarming challenge in the treatment of these opportunistic bacteria (19). Although enterobacter strains have full resistance to cefepime and ceftriaxone, their sensivity to amikacin is about 53.8%. Meropenem with the sensitivity of 75% to various enterobacters and imipenem with sensitivity of 62.5% to various enterobacters which had the highest efficacy on them. While in the study conducted by Alikhani in the same center in 2008-09, the sensitivity of species of enterobacters to meropenem was 85.5% and to imipenem and amikacin was 71.5% (18). It is evident that carbapenems continue to be suitable drugs against enterobacter. However, increased resistance made to these two widely-used antibiotics is an alarm in the treatment of nosocomial infections. 

In conclusion, the present study showed that the resistance of different organisms to cephalosporins increases considerably. On the other hand, there is still desirable sensitivity to the family of carbapenems among them Gram-negative bacteria. The small number of cases of this study was its weakness and we need other studies with more cases and also it necessary conducting such a study elsewhere. 

## Notes

**Funding:** This project was financially supported by the Research Chancellery of Mazandaran University of Medical Sciences.
